# Gel immersion endoscopic mucosal resection in early gastric cancer with bleeding: A case report

**DOI:** 10.1016/j.amsu.2022.104743

**Published:** 2022-09-21

**Authors:** Daisuke Suto, Masashi Yoshida, Takaaki Otake, Yosuke Osawa, Takayuki Akita, Kiichi Sato, Hidehiko Yamada, Hironori Odaira, Yutaka Suzuki, Yutaka Kohgo

**Affiliations:** a Department of Gastroenterology, International University of Health and Welfare Hospital, 537-3 Iguchi, Nasushiobara, Tochigi, 329-2763, Japan; b Department of Surgery, International University of Health and Welfare Hospital, 537-3, Iguchi, Nasushiobara, Tochigi, 329-2763, Japan

**Keywords:** Bleeding, Chronic renal failure, Early gastric cancer, Endoscopic mucosal resection, Gel immersion

## Abstract

Gel immersion endoscopy was developed by Yano for the treatment of bleeding. In this case, we performed gel immersion endoscopic mucosal resection to treat a bleeding gastric cancer. An 80-year-old man, with chronic renal failure and on aspirin treatment for ischemic heart disease, underwent endoscopic treatment for multiple early gastric cancers on the anterior and posterior walls of the pyloric ring. An endoscopic submucosal dissection was performed for gastric cancer on the anterior wall; however, the removal of the cancer on the posterior wall was complicated by tumor prolapse and bleeding. Gel formulation (VISCOCLEAR® Otsuka Pharmaceutical Factory, Inc., Tokushima, Japan) was used to immerse the bleeding tumor and subsequently facilitate the endoscopic mucosal resection. Various factors, such as the use of antithrombotic medication and underlying renal disease, can increase the risk of bleeding during endoscopic gastric cancer resection. If bleeding persists, the resection margin becomes obscured. Gel formulations, such as VISCOCLEAR®, can be applied to control bleeding and improve visibility. In this case, gel immersion was useful for endoscopic mucosal resection of the bleeding tumor. The use of gel immersion endoscopic resection should be considered for the treatment of early gastric cancer, however further cases should be evaluated.

## Highlights

• 80-year-old man with bleeding from oral antithrombotic medication and early gastric cancer.
• A new formulation of Viscoclear gel successfully prevented loss of visibility due to bleeding.
• Successful identification of the resection line of early-stage gastric cancer with the use of Viscoclear.
• Good visual field was secured with the use of Viscoclear.
• Post endoscopic treatment course was uneventful.

## 1 Introduction

Gel immersion endoscopy was first reported by Yano et al. as a good therapeutic option for hemorrhage [1]. The gel formulation, launched as VISCOCLEAR® (VISCOCLEAR®, Otsuka Pharmaceutical Factory, Inc., Tokushima, Japan), prevents profuse bleeding and makes it easier to identify the site of bleeding. Later, endoscopic submucosal dissection (ESD) and endoscopic submucosal resection (EMR) using VISCOCLEAR® [2] were reported to be helpful [3]. From this, we can speculate that gel immersion mucosal resection could be useful for treating hemorrhagic gastric tumors. Here, we report a case of a patient with bleeding gastric cancer who was undergoing dialysis and percutaneous coronary intervention and was taking aspirin. In this case, we performed gel immersion mucosal resection of hemorrhagic gastric tumors. The case has been reported in line with the SCARE checklist [4].

## 2 Presentation of case

**Fig. 1:**
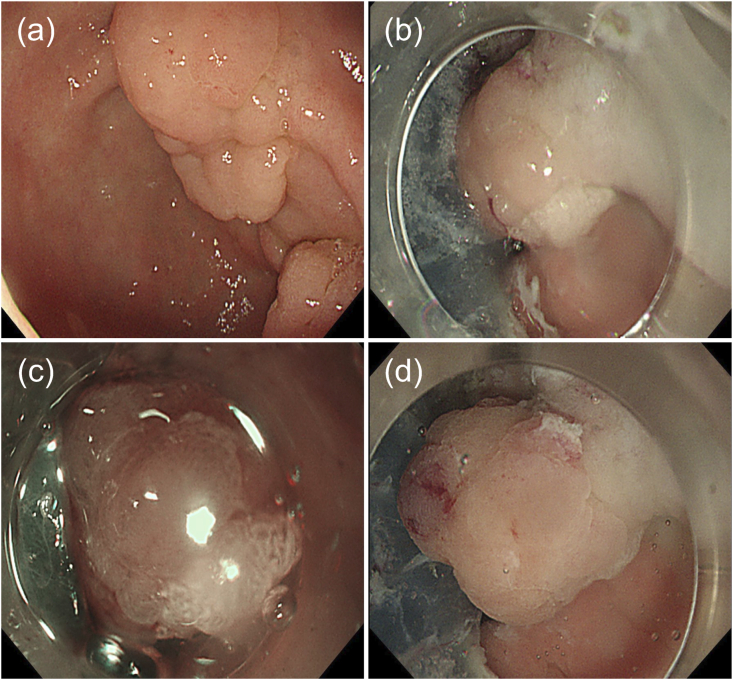
(a) Two lesions of early gastric cancer near the pyloric ring. (b) Bleeding from the tumor was observed gradually. (c) Obtaining a field of view for endoscopic submucosal dissection was difficult owing to bleeding from the tumor and prolapse into the duodenum. (d) Injection of the gel formulation provided a clear field of vision by preventing tumor prolapse into the duodenum.

**Fig. 2:**
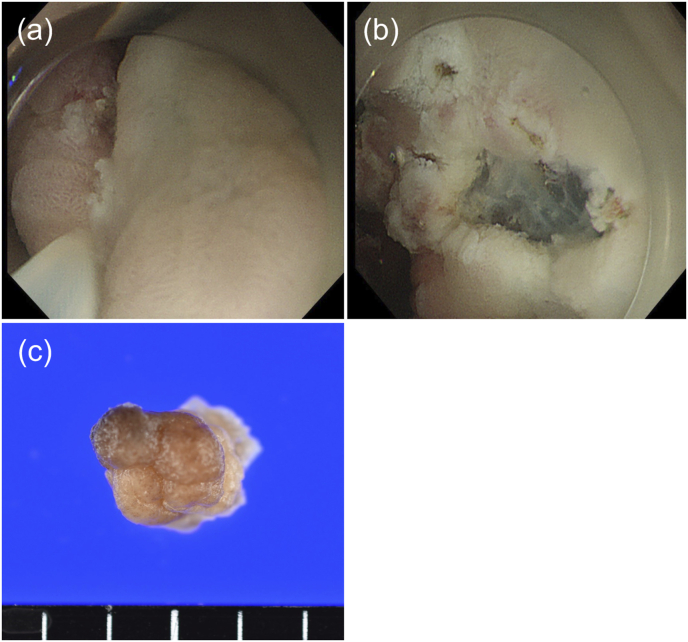
The stomach was filled with a gel formulation to obtain a clear view. (a) The tumor's boundaries were identified, and the snare was then strangulated. (b) After gel immersion resection. (c) Post-endoscopic resection specimen.

**Fig. 3:**
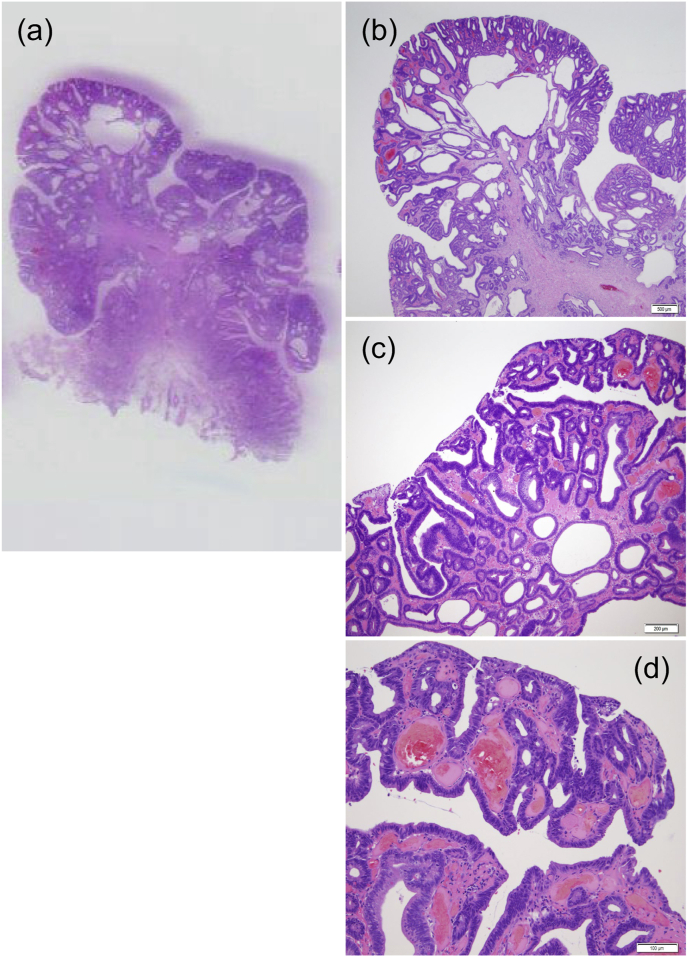
(a) The loupe image is shown. (b) Localized tumor cells in the mucosa (Scale bar: 500 μm). (c) Localized tumor cells in the mucosa (Scale bar: 200 μm) (d) Localized tumor cells in the mucosa (Scale bar: 100 μm).

An 80-year-old man was on aspirin for ischemic heart disease and was undergoing dialysis for chronic renal failure. An esophagogastroduodenoscopy was performed to locate the site of gastrointestinal bleeding to treat the patient's anemia. A 30-mm whitish elevated type IIa tumor on the anterior wall of the pyloric ring and a 12-mm erythematous type 0-I tumor on the posterior wall were detected (Fig. 1a). These tumors were diagnosed as adenocarcinomas (tub1) by biopsies. ESD was performed for the gastric cancer on the anterior wall. Subsequently, we intended to perform ESD for the tumor on the posterior wall, but the tumor began to prolapse into the duodenum because of peristalsis (Fig. 1b). Furthermore, it started to bleed, resulting in poor visibility (Fig. 1c). We injected VISCOCLEAR® into the stomach to identify the bleeding site. The gel formulation pooled around the lesion, providing sufficient floating force and preventing further tumor prolapse into the duodenum (Fig. 1d). The bleeding point was detected to be at the top of the tumor. The lesion margins were observed (Fig. 2a), and snaring was easily performed within 2 min (Fig. 2b). Histopathological examination revealed that the tumor was a highly differentiated intramucosal adenocarcinoma with negative vertical and lateral margins (Figs. 2c, Fig. 3a–d). A follow-up esophagogastroduodenoscopy is scheduled at 2 months after this endoscopic treatment. If this follow-up remains uneventful, another esophagogastroduodenoscopy will be performed 1 year later.

## 3 Discussion

The prevalence of chronic renal failure is increasing worldwide, and the risk of incidental gastric cancer is significantly higher in patients with chronic renal failure than in the general population [5]. Patients with chronic renal failure, such as those on hemodialysis, have a 13.5–33.3% risk of bleeding after gastric ESD [6]. Furthermore, the proportion of patients with gastric cancer receiving antithrombotic medication has been increasing with age. Patients on aspirin alone, who are at a high risk of thromboembolism, can undergo the procedure without drug discontinuation [7]. In this case, the patient was undergoing dialysis, and the risk of rebleeding from the tumor was high because the patient continued to take antithrombotic medication; the tumor was expected to be dislodged into the duodenum by peristalsis. We were successful in performing gel immersion EMR in a patient with multiple risk factors for gastric bleeding.

In a previously reported case of multiple diverticular hemorrhages, endoscopic hemostasis using VISCOCLEAR® helped to identify the guttural vessels with good visibility [8]. In addition, gel formulation has been reported to be useful in cases of bleeding from duodenal ulcers [9]. Recently, EMR of early gastric cancer without bleeding near the pyloric ring using the gel immersion technique has been reported [3]. In our case, the patient's tumor was bleeding and visibility was poor. This prompted the need for gel immersion, which not only aided in identifying the dissection line but also prevented the tumor from prolapsing back into the duodenum. We had considered filling the stomach with water, since underwater EMR is an immersion technique with a high resection rate and low incidence of adverse events. However, water usually flows into the duodenum, making it difficult to keep the water in the stomach [10]. By filling the area around the tumor with gel, the buoyancy force prevented the tumor from prolapsing into the duodenum, and the boundary of the tumor became clear, allowing easy snaring. We believe that filling the stomach with a gel benefitted the procedure in terms of easy access, and we intend to study more of these cases in the future.

## 4 Conclusion

In this case, we found that the gel immersion method was useful for treating early gastric cancer with bleeding from the tumor. When endoscopic treatment of gastric cancer is difficult because of poor visibility caused by bleeding, the gel immersion method should be considered as one of the treatment options. The effectiveness of gel immersion endoscopic mucosal resection needs to be evaluated in more such cases in the future.

## Ethical approval

The study was approved by the Ethics Committee of the International University of Health and Welfare Hospital [approval number: 22-B-17].

## Source of funding

None.

## Author contribution

All authors contributed equally to the manuscript.

## Trail registry number

Name of the registry: This paper is case report. The authors don't need to register this work.

## Garantor

Daisuke Suto, First Author. Masashi Yoshida, Senior Author.

## Funding sources

None.

## Consent of patient

Written informed consent was obtained from the patient to publish this case report and any accompanying images.

## Declaration of competing interest

None.
